# Supplementary material for: IWS1 positions downstream DNA to globally stimulate Pol II elongation
Source: Nat Commun. 2025 Aug 20;16:7747. doi: 10.1038/s41467-025-62913-5 (PMC12368070; doi:10.1038/s41467-025-62913-5)
Supplement: Supplementary file 2 — Description of Additional Supplementary Files [file 41467_2025_62913_MOESM2_ESM.pdf]

Description of Additional Supplementary Files

Supplementary Data 1. Chromatin proteomics mass spectrometry quantitative data after 1 h and 4 h IWS1 depletions. p-values were assessed using two-sided Student's t-test.

Supplementary Data 2. Raw quantification data for RNA extension assays.
