# Supplementary material for: IWS1 positions downstream DNA to globally stimulate Pol II elongation
Source: Nat Commun. 2025 Aug 20;16:7747. doi: 10.1038/s41467-025-62913-5 (PMC12368070; doi:10.1038/s41467-025-62913-5)
Supplement: Supplementary file 5 — Reporting Summary [file 41467_2025_62913_MOESM5_ESM.pdf]

nature portfolio

Corresponding author(s): Patrick Cramer, Kristina Zumer

Last updated by author(s): Jul 29, 2025

Reporting Summary

Nature Portfolio wishes to improve the reproducibility of the work that we publish. This form provides structure for consistency and transparency in reporting. For further information on Nature Portfolio policies, see our Editorial Policies and the Editorial Policy Checklist.

Statistics

For all statistical analyses, confirm that the following items are present in the figure legend, table legend, main text, or Methods section.

n/a	Confirmed	
☐	☒	The exact sample size (n) for each experimental group/condition, given as a discrete number and unit of measurement
☐	☒	A statement on whether measurements were taken from distinct samples or whether the same sample was measured repeatedly
☐	☒	The statistical test(s) used AND whether they are one- or two-sided<br>*Only common tests should be described solely by name; describe more complex techniques in the Methods section.*
☒	☐	A description of all covariates tested
☐	☒	A description of any assumptions or corrections, such as tests of normality and adjustment for multiple comparisons
☐	☒	A full description of the statistical parameters including central tendency (e.g. means) or other basic estimates (e.g. regression coefficient) AND variation (e.g. standard deviation) or associated estimates of uncertainty (e.g. confidence intervals)
☐	☒	For null hypothesis testing, the test statistic (e.g. F, t, r) with confidence intervals, effect sizes, degrees of freedom and P value noted<br>*Give P values as exact values whenever suitable.*
☒	☐	For Bayesian analysis, information on the choice of priors and Markov chain Monte Carlo settings
☒	☐	For hierarchical and complex designs, identification of the appropriate level for tests and full reporting of outcomes
☐	☒	Estimates of effect sizes (e.g. Cohen's d, Pearson's r), indicating how they were calculated

Our web collection on statistics for biologists contains articles on many of the points above.

Software and code

Policy information about availability of computer code

Data collection	Genome-wide sequencing data were collected using Illumina NextSeq 550.

Data analysis

TT-seq reads were aligned to the human reference genome (GRCh38) that had been augmented with synthetic RNA spike-in sequences. We utilized STAR version 2.6.0 for alignment with the following parameters: --runThreadN 24 --readFilesCommand zcat --outFilterType BySJout --outFilterMultimapNmax 1 --alignSJoverhangMin 8 --alignSJDBoverhangMin 1 --outFilterMismatchNmax 999 --outFilterMismatchNoverLmax 0.02 --alignIntronMin 20 --alignIntronMax 1000000 --alignMatesGapMax 1000000.
For mNET-seq, the alignment was performed against a composite reference consisting of the human GRCh38 genome and the S. cerevisiae SacCer3 genome, using STAR version 2.6.0 with parameters: --runThreadN 24 --readFilesCommand zcat --outFilterType BySJout --outFilterMultimapNmax 1 --outFilterMismatchNoverLmax 0.02 --outFilterMatchNmin 16 --outFilterScoreMinOverLread 0 --outFilterMatchNminOverLread 0 --alignIntronMax 500000.
For TT-seq and mNET-seq, the quantifications of reads aligning to the spike-in sequences were used to calculate normalization factors, ensuring accurate comparison of expression levels across samples as in (28). Spike-in read counts were assessed by summarizeOverlaps function (Bioconductor package "IRanges") and normalization factors were calculated with DESeq2.
ChIP-seq reads were aligned to a hybrid reference combining the human GRCh38 genome and the D. melanogaster genome, employing Bowtie2 version 2.3.4.1 with options: -p 24 --no-discordant --no-mixed --very-sensitive. Picard tools (http://broadinstitute.github.io/picard) were used to remove the duplicates. ChIP-seq data was normalized to the coverage at non-transcribed regions. ChIP-seq counts at non-transcribed regions were assessed using summarizeOverlaps function (Bioconductor package "IRanges") and normalization factors were calculated with DESeq2.
All downstream analyses were conducted in RStudio, using R version 4.3.1. The analysis relied on packages sourced from the Bioconductor repository and the Tidyverse suite , with graphical representations generated through the ggplot2 package.

For manuscripts utilizing custom algorithms or software that are central to the research but not yet described in published literature, software must be made available to editors and reviewers. We strongly encourage code deposition in a community repository (e.g. GitHub). See the Nature Portfolio guidelines for submitting code & software for further information.

Data

Policy information about availability of data

All manuscripts must include a data availability statement. This statement should provide the following information, where applicable:

- Accession codes, unique identifiers, or web links for publicly available datasets
- A description of any restrictions on data availability
- For clinical datasets or third party data, please ensure that the statement adheres to our policy

NGS datasets generated in this study have been deposited in NCBI's Gene Expression Omnibus database (GEO) and are accessible through GEO Series accession numbers GSE276547, GSE276549, GSE276550. The mass spectrometry proteomics data have been deposited to the ProteomeXchange Consortium via the PRIDE (92) partner repository with the dataset identifier PXD056248 and PXD066658.
The cryo-EM reconstructions corresponding to MAPS 1–12 have been deposited in the Electron Microscopy Data Bank under accession codes EMD-54196, EMD-54197, EMD-54201, EMD-54202, EMD-54203, EMD-54204, EMD-54205, EMD-54206, EMD-54207, EMD-54208, EMD-54209, EMD-54210, EMD-54211, and EMD-54212. The atomic model of the activated RNA polymerase II elongation complex bound to IWS1 and ELOF1 has been deposited in the Protein Data Bank under accession code 9RTT.

Research involving human participants, their data, or biological material

Policy information about studies with human participants or human data. See also policy information about sex, gender (identity/presentation), and sexual orientation and race, ethnicity and racism.

Reporting on sex and gender	Not applicable
Reporting on race, ethnicity, or other socially relevant groupings	Not applicable
Population characteristics	Not applicable
Recruitment	Not applicable
Ethics oversight	Not applicable

Note that full information on the approval of the study protocol must also be provided in the manuscript.

Field-specific reporting

Please select the one below that is the best fit for your research. If you are not sure, read the appropriate sections before making your selection.

[x] Life sciences [ ] Behavioural & social sciences [ ] Ecological, evolutionary & environmental sciences

For a reference copy of the document with all sections, see nature.com/documents/nr-reporting-summary-flat.pdf

Life sciences study design

All studies must disclose on these points even when the disclosure is negative.

Sample size	All information regarding sample sizes used in each analysis is clearly stated in the Methods and/or Results sections of this study. No explicit power analysis was performed. Sample size of each measurement was determined by the practical limitations of the protocol utilized.
Data exclusions	All criteria for exclusion or inclusion of data points (genes used in the analysis) are clearly stated in the Methods and/or Results section of this study. Outliers were removed from boxplots for clearer visualization.
Replication	TT-seq, mNET-seq, ChIP-seq experiments were performed in 2 biological replicates. Chromatin proteomics was performed in 4 biological replicates. In vitro RNA extension assay reactions were performed in quadruplicate.
Randomization	Different stocks were opened and cultured separately for several passages. On the day of the experiment, plates were randomly assigned to replicates to ensure unbiased sample distribution.
Blinding	Not applicable

Reporting for specific materials, systems and methods

We require information from authors about some types of materials, experimental systems and methods used in many studies. Here, indicate whether each material, system or method listed is relevant to your study. If you are not sure if a list item applies to your research, read the appropriate section before selecting a response.

Materials & experimental systems

n/a	Involved in the study
☐	☒ Antibodies
☐	☒ Eukaryotic cell lines
☒	☐ Palaeontology and archaeology
☒	☐ Animals and other organisms
☒	☐ Clinical data
☒	☐ Dual use research of concern
☒	☐ Plants

Methods

n/a	Involved in the study
☐	☒ ChIP-seq
☒	☐ Flow cytometry
☒	☐ MRI-based neuroimaging

Antibodies

Antibodies used	anti-HA (Roche, REF: 11867431001), anti-IWS1 (Proteintech, REF: 16943-1-AP), anti-H3 (Abcam, REF: ab21054), anti-U1-snRNP70 (SCBT, REF: sc-390899), Rpb1 NTD (Cell Signaling Technology, REF: D8L4Y), Histone H3K36me3 (Active Motif, REF: 61021), RNA Polymerase II antibody (Diagenode, REF: C15200004)
Validation	Validations of the antibodies were performed by the manufacturers.

Eukaryotic cell lines

Policy information about cell lines and Sex and Gender in Research

Cell line source(s)	K562 (DSMZ, ACC 10), K562 IWS1-dTAG (This study), S. cerevisiae strain BY4741 (Euroscarf, ACC-Y00000), D. melanogaster S2 cell line (DSMZ, ACC 130), K562 RTF1-dTAG (Zumer et al., 2021; PMID: 34146481), K562 SPT6-dTAG (Zumer et al., 2021; PMID: 34146481)
Authentication	K562 IWS1-dTAG was validated by western blotting, using an HA epitope-specific and IWS1-specific antibodies to detect the dTAG, and Sanger sequencing of the PCR-amplified integration site.
Mycoplasma contamination	The cells in culture were checked for mycoplasma quarterly with the PlasmoTest kit (InvivoGen) kit and are mycoplasma negative.
Commonly misidentified lines (See ICLAC register)	No commonly misidentified cell lines were used.

Plants

Seed stocks

Report on the source of all seed stocks or other plant material used. If applicable, state the seed stock centre and catalogue number. If plant specimens were collected from the field, describe the collection location, date and sampling procedures.

Novel plant genotypes

Describe the methods by which all novel plant genotypes were produced. This includes those generated by transgenic approaches, gene editing, chemical/radiation-based mutagenesis and hybridization. For transgenic lines, describe the transformation method, the number of independent lines analyzed and the generation upon which experiments were performed. For gene-edited lines, describe the editor used, the endogenous sequence targeted for editing, the targeting guide RNA sequence (if applicable) and how the editor was applied.

Authentication

Describe any authentication procedures for each seed stock used or novel genotype generated. Describe any experiments used to assess the effect of a mutation and, where applicable, how potential secondary effects (e.g. second site T-DNA insertions, mosiacism, off-target gene editing) were examined.

ChIP-seq

Data deposition

☒ Confirm that both raw and final processed data have been deposited in a public database such as GEO.

☐ Confirm that you have deposited or provided access to graph files (e.g. BED files) for the called peaks.

Data access links
May remain private before publication.

NGS datasets generated in this study have been deposited in NCBI's Gene Expression Omnibus database (GEO) and are accessible through GEO Series accession numbers GSE276547, GSE276549, GSE276550.

Files in database submission

IWS1_chip_H3K36me3_r1_DMSO_1h_S5_R1_001.fastq.gz
IWS1_chip_H3K36me3_r1_DMSO_1h_S5_R2_001.fastq.gz
IWS1_chip_H3K36me3_r1_DMSO_4h_S5_R1_001.fastq.gz
IWS1_chip_H3K36me3_r1_DMSO_4h_S5_R2_001.fastq.gz
IWS1_chip_H3K36me3_r1_DTAG_1h_S6_R1_001.fastq.gz
IWS1_chip_H3K36me3_r1_DTAG_1h_S6_R2_001.fastq.gz
IWS1_chip_H3K36me3_r1_DTAG_4h_S6_R1_001.fastq.gz
IWS1_chip_H3K36me3_r1_DTAG_4h_S6_R2_001.fastq.gz
IWS1_chip_H3K36me3_r2_DMSO_1h_S3_R1_001.fastq.gz
IWS1_chip_H3K36me3_r2_DMSO_1h_S3_R2_001.fastq.gz
IWS1_chip_H3K36me3_r2_DMSO_4h_S3_R1_001.fastq.gz
IWS1_chip_H3K36me3_r2_DMSO_4h_S3_R2_001.fastq.gz
IWS1_chip_H3K36me3_r2_DTAG_1h_S4_R1_001.fastq.gz
IWS1_chip_H3K36me3_r2_DTAG_1h_S4_R2_001.fastq.gz
IWS1_chip_H3K36me3_r2_DTAG_4h_S4_R1_001.fastq.gz
IWS1_chip_H3K36me3_r2_DTAG_4h_S4_R2_001.fastq.gz
IWS1_r1_DMSO_1h_S1_R1_001.fastq.gz
IWS1_r1_DMSO_1h_S1_R2_001.fastq.gz
IWS1_r1_DTAG_1h_S2_R1_001.fastq.gz
IWS1_r1_DTAG_1h_S2_R2_001.fastq.gz
IWS1_r2_DMSO_1h_S3_R1_001.fastq.gz
IWS1_r2_DMSO_1h_S3_R2_001.fastq.gz
IWS1_r2_DTAG_1h_S4_R1_001.fastq.gz
IWS1_r2_DTAG_1h_S4_R2_001.fastq.gz
L_IWS1_r1_DMSO_1h_S1_R1_001.fastq.gz
L_IWS1_r1_DMSO_1h_S1_R2_001.fastq.gz
L_IWS1_r1_DTAG_1h_S2_R1_001.fastq.gz
L_IWS1_r1_DTAG_1h_S2_R2_001.fastq.gz
L_IWS1_r2_DMSO_1h_S3_R1_001.fastq.gz
L_IWS1_r2_DMSO_1h_S3_R2_001.fastq.gz
L_IWS1_r2_DTAG_1h_S4_R1_001.fastq.gz
L_IWS1_r2_DTAG_1h_S4_R2_001.fastq.gz
PolII_chip_IWS1_r1_DMSO_1h_S13_R1_001.fastq.gz
PolII_chip_IWS1_r1_DMSO_1h_S13_R2_001.fastq.gz
PolII_chip_IWS1_r1_DMSO_1h_S1_R1_001.fastq.gz
PolII_chip_IWS1_r1_DMSO_1h_S1_R2_001.fastq.gz
PolII_chip_IWS1_r1_DTAG_1h_S14_R1_001.fastq.gz
PolII_chip_IWS1_r1_DTAG_1h_S14_R2_001.fastq.gz
PolII_chip_IWS1_r1_DTAG_1h_S2_R1_001.fastq.gz
PolII_chip_IWS1_r1_DTAG_1h_S2_R2_001.fastq.gz
PolII_chip_IWS1_r2_DMSO_1h_S15_R1_001.fastq.gz
PolII_chip_IWS1_r2_DMSO_1h_S15_R2_001.fastq.gz
PolII_chip_IWS1_r2_DMSO_1h_S3_R1_001.fastq.gz

PolII_chip_IWS1_r2_DMSO_1h_S3_R2_001.fastq.gz
PolII_chip_IWS1_r2_DTAG_1h_S16_R1_001.fastq.gz
PolII_chip_IWS1_r2_DTAG_1h_S16_R2_001.fastq.gz
PolII_chip_IWS1_r2_DTAG_1h_S4_R1_001.fastq.gz
PolII_chip_IWS1_r2_DTAG_1h_S4_R2_001.fastq.gz
IWS1_chip_H3K36me3_r1_DMSO_1h.norm.coverage.bigWig
IWS1_chip_H3K36me3_r1_DMSO_4h.norm.coverage.bigWig
IWS1_chip_H3K36me3_r1_DTAG_1h.norm.coverage.bigWig
IWS1_chip_H3K36me3_r1_DTAG_4h.norm.coverage.bigWig
IWS1_chip_H3K36me3_r2_DMSO_1h.norm.coverage.bigWig
IWS1_chip_H3K36me3_r2_DMSO_4h.norm.coverage.bigWig
IWS1_chip_H3K36me3_r2_DTAG_1h.norm.coverage.bigWig
IWS1_chip_H3K36me3_r2_DTAG_4h.norm.coverage.bigWig
IWS1_r1_DMSO_1h.norm.coverage.minus.bigWig
IWS1_r1_DMSO_1h.norm.coverage.plus.bigWig
IWS1_r1_DTAG_1h.norm.coverage.minus.bigWig
IWS1_r1_DTAG_1h.norm.coverage.plus.bigWig
IWS1_r2_DMSO_1h.norm.coverage.minus.bigWig
IWS1_r2_DMSO_1h.norm.coverage.plus.bigWig
IWS1_r2_DTAG_1h.norm.coverage.minus.bigWig
IWS1_r2_DTAG_1h.norm.coverage.plus.bigWig
L_IWS1_r1_DMSO_1h.norm.coverage.minus.bigWig
L_IWS1_r1_DMSO_1h.norm.coverage.plus.bigWig
L_IWS1_r1_DTAG_1h.norm.coverage.minus.bigWig
L_IWS1_r1_DTAG_1h.norm.coverage.plus.bigWig
L_IWS1_r2_DMSO_1h.norm.coverage.minus.bigWig
L_IWS1_r2_DMSO_1h.norm.coverage.plus.bigWig
L_IWS1_r2_DTAG_1h.norm.coverage.minus.bigWig
L_IWS1_r2_DTAG_1h.norm.coverage.plus.bigWig
PolII_chip_SSRP1_r1_DMSO_1h.norm.coverage.bigWig
PolII_chip_SSRP1_r1_DTAG_1h.norm.coverage.bigWig
PolII_chip_SSRP1_r2_DMSO_1h.norm.coverage.bigWig
PolII_chip_SSRP1_r2_DTAG_1h.norm.coverage.bigWig
H3K36me3_chip_RTF1_r1_DMSO_1h_S9_R1_001.fastq.gz

Genome browser session (e.g. UCSC)

Not applicable

Methodology

Replicates

All ChIp-seq experiments were performed in 2 biological replicates.

Sequencing depth

All ChIP-seq libraries were sequenced paired-end using 43 cycles on the NextSeq550 (Illumina) platform.

Antibodies

Histone H3K36me3 antibody (Active Motif, 61021), Rpb1 NTD antibody (Cell Signaling Technology, 14958)

Peak calling parameters

N/A

Data quality

Data quality was ensured by enforcing a minimum cut-off based on the read count distribution to be used for analysis and making inferences.

Software

ChIP-seq reads were aligned to a hybrid reference combining the human GRCh38 genome and the D. melanogaster genome, employing Bowtie2 version 2.3.4.1 with options: -p 24 --no-discordant --no-mixed --very-sensitive. Picard tools (http://broadinstitute.github.io/picard) were used to remove the duplicates. ChIP-seq data was normalized to the coverage at non-transcribed regions. ChIP-seq counts at non-transcribed regions were assessed using summarizeOverlaps function (Bioconductor package "IRanges") and normalization factors were calculated with DESeq2.
