# Supplementary figures and images for: IWS1 positions downstream DNA to globally stimulate Pol II elongation
Source: Nat Commun. 2025 Aug 20;16:7747. doi: 10.1038/s41467-025-62913-5 (PMC12368070; doi:10.1038/s41467-025-62913-5)

Source Data Fig. 1b

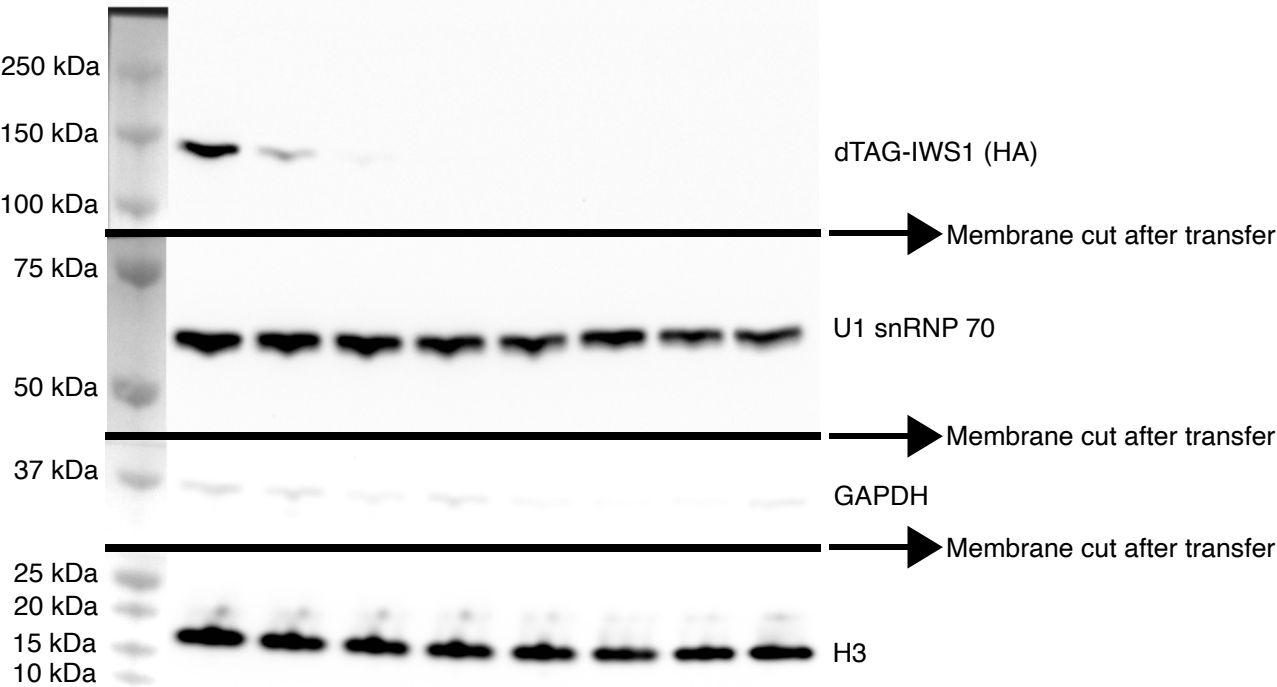

Source Data Supplementary Fig. 1b

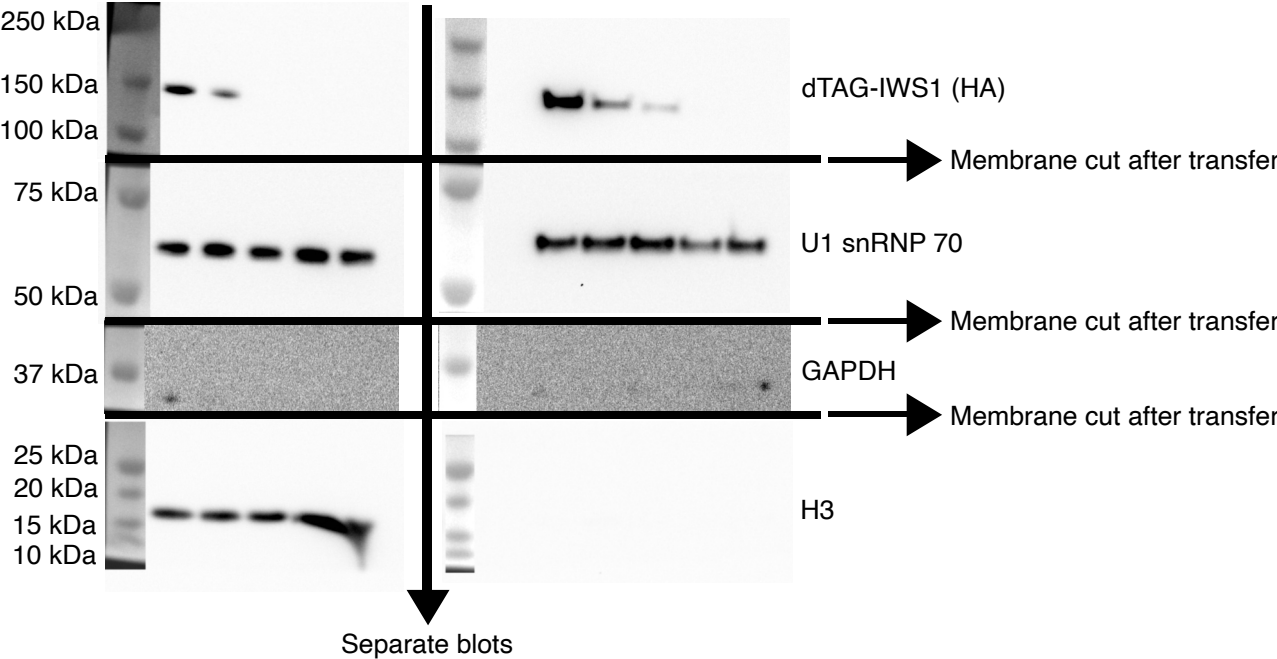

Source Data Supplementary Fig. 1c

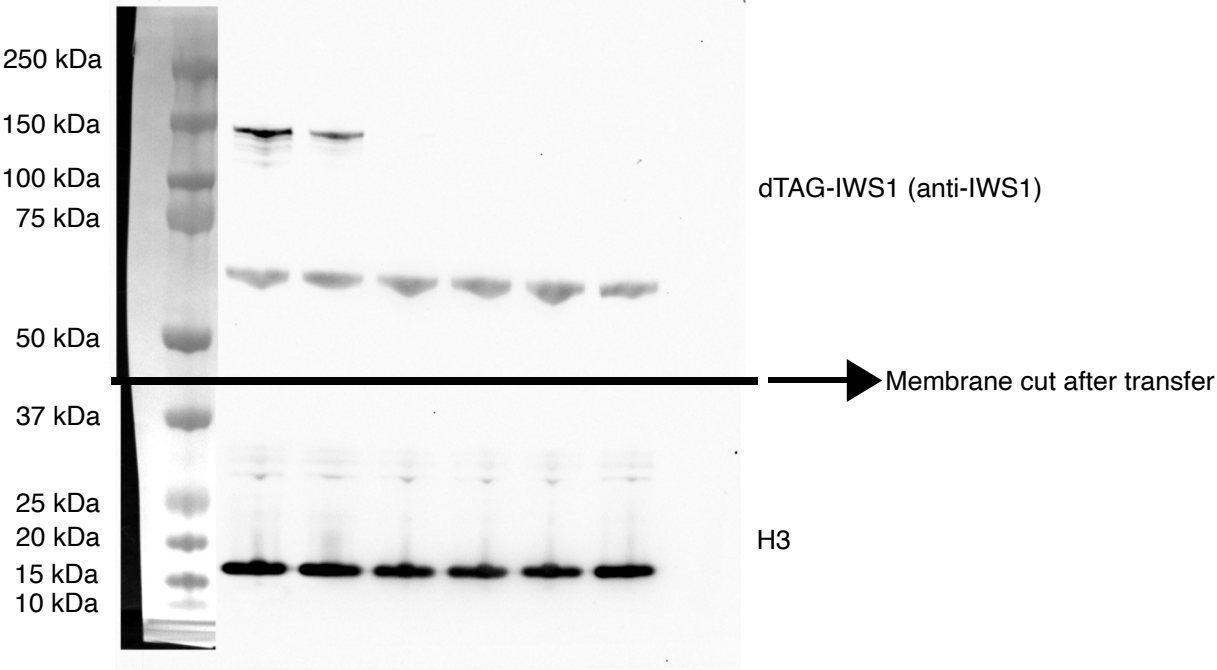

Supplement: Supplementary file 7 — Source Data [file 41467_2025_62913_MOESM7_ESM.zip › IWS1_Source_Data_Fig1b_Suppl_Fig1b_1c.pdf]
