# Supplementary material for: IWS1 positions downstream DNA to globally stimulate Pol II elongation
Source: Nat Commun. 2025 Aug 20;16:7747. doi: 10.1038/s41467-025-62913-5 (PMC12368070; doi:10.1038/s41467-025-62913-5)
Supplement: Supplementary file 1 — Supplementary Information [file 41467_2025_62913_MOESM1_ESM.pdf]

IWS1 positions downstream DNA to globally stimulate Pol II elongation

Aiturgan Zheenbekova[1*], James L. Walshe[1*], Moritz Ochmann[1], Moritz Bäuerle[1], Ute Neef[1], Kerstin C. Maier[1], Petra Rus[1], Yumeng Yan[2], Henning Urlaub[2], Patrick Cramer[1#], Kristina Žumer[1#]

Supplementary Figures 1 - 11

Supplementary Tables 1, 2

[1]Department of Molecular Biology, Max Planck Institute for Multidisciplinary Sciences, Am Faßberg 11, 37077 Göttingen, Germany.

[2]Bioanalytical Mass Spectrometry, Max Planck Institute for Multidisciplinary Sciences, Am Faßberg 11, 37077 Göttingen, Germany.

[#]Correspondence: patrick.cramer@mpinat.mpg.de, kristina.zumer@mpinat.mpg.de

*These authors contributed equally

Supplementary Fig. 1

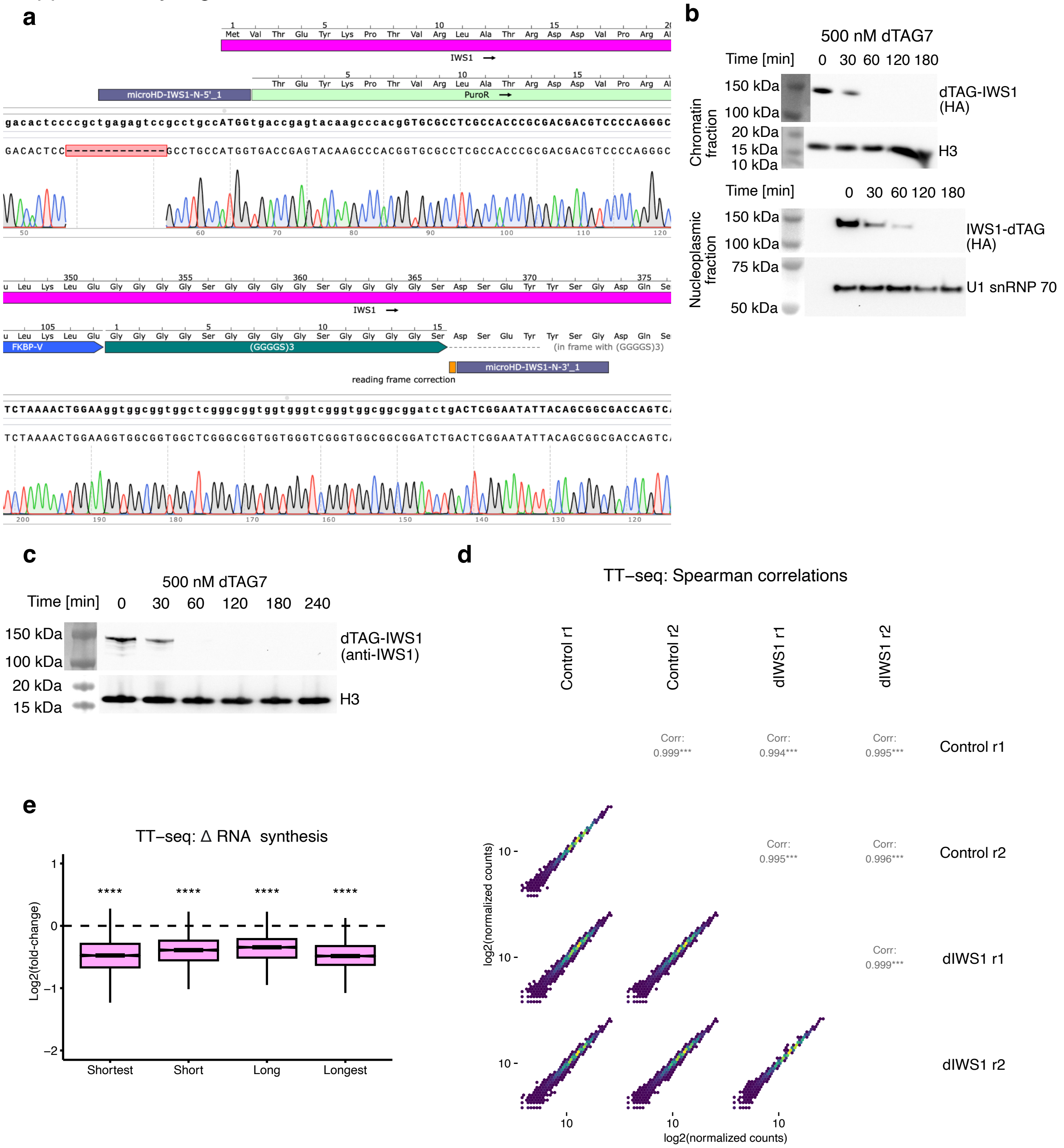

Supplementary Fig. 1. IWS1 depletion leads to decreased RNA synthesis.

a) Validation of the dTAG insertion into IWS1 gene by Sanger sequencing of genomic DNA.
b) Western blot analysis of IWS1 (anti-HA) in chromatin and nucleoplasmic fractions of cells treated with dTAG7 for the indicated time. H3 and U1 snRNP 70 are loading controls.
c) Western blot analysis of IWS1 (anti-IWS1) in lysates of dTAG-IWS1 cells treated with dTAG7 for the indicated time. U1 snRNP 70 is a loading control.
d) Scatter plots of normalized TT-seq gene counts and Spearman correlation coefficients (rho) of all sample pairs (n=10147).
e) Boxplots showing changes in RNA synthesis (TT-seq) of genes grouped based on gene lengths: shortest (< 10 kb, n=2537), short (10–26.5 kb, n=2537), long (26.5–65 kb, n=2536), and longest (> 65 kb, n=2537) genes. Representation as in Fig. 1c. Shortest: p-value < $2.2*10^{-16}$, short: p-value < $2.2*10^{-16}$, long: p-value < $2.2*10^{-16}$, longest: p-value < $2.2*10^{-16}$.

Supplementary Fig. 2

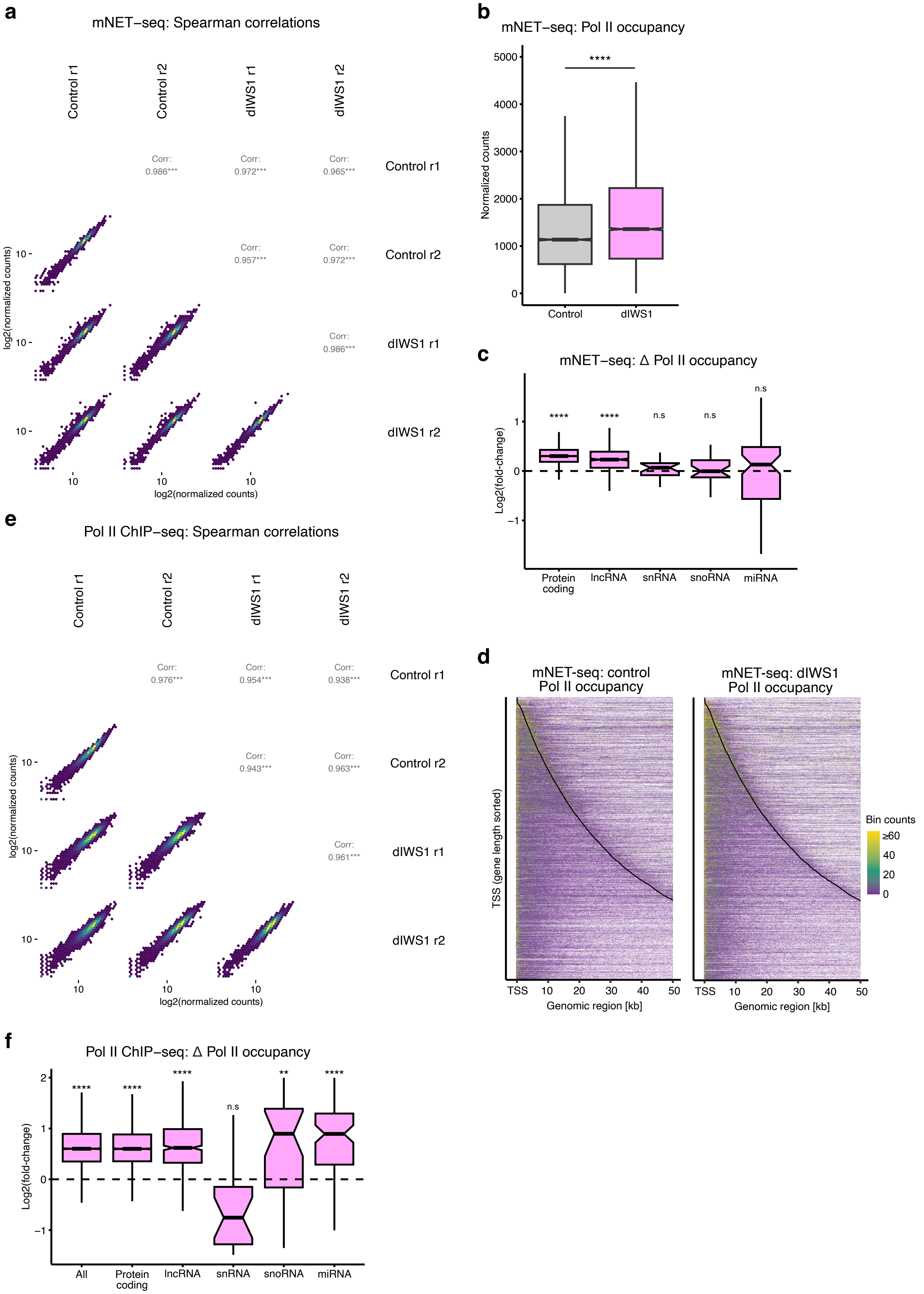

Supplementary Fig. 2

Supplementary Fig. 2. Loss of IWS1 decreases Pol II elongation velocity.

a) Scatter plots of normalized mNET-seq gene counts and Spearman correlation coefficients (rho) of all sample pairs (n=10147).
b) Boxplots showing mNET-seq counts of expressed genes (n=10147) in 1 h control- or dTAG7-treated (dIWS1) cells. Representation as in Fig. 2e. p-value $< 2.2*10^{-16}$.
c) Boxplots showing changes in Pol II occupancy (mNET-seq) for individual gene biotypes (protein-coding, n=9089; lncRNA, n=615; snRNA, n=25; snoRNA, n=41; miRNA, n=145). Representation as in Fig. 1c. Protein-coding: p-value $< 2.2*10^{-16}$, lncRNA: p-value $< 2.2*10^{-16}$, snRNA: p-value = 0.187, snoRNA: p-value = 0.7451, miRNA: p-value = 0.3485.
d) Heatmap representation of mNET-seq signal of expressed genes (n=10147) in 1 h control- or dTAG7-treated (dIWS1) cells. Representation as in Fig. 2b.
e) Scatter plots of normalized Pol II ChIP-seq gene counts and Spearman correlation coefficients (rho) of all sample pairs (n=10147).
f) As in c), but for Pol II ChIP-seq. All: p-value $< 2.2*10^{-16}$, protein-coding: p-value $< 2.2*10^{-16}$, lncRNA: p-value $< 2.2*10^{-16}$, snRNA: p-value = 0.147, snoRNA: p-value = 0.005829, miRNA: p-value = $5.173*10^{-14}$.

Supplementary Fig. 3

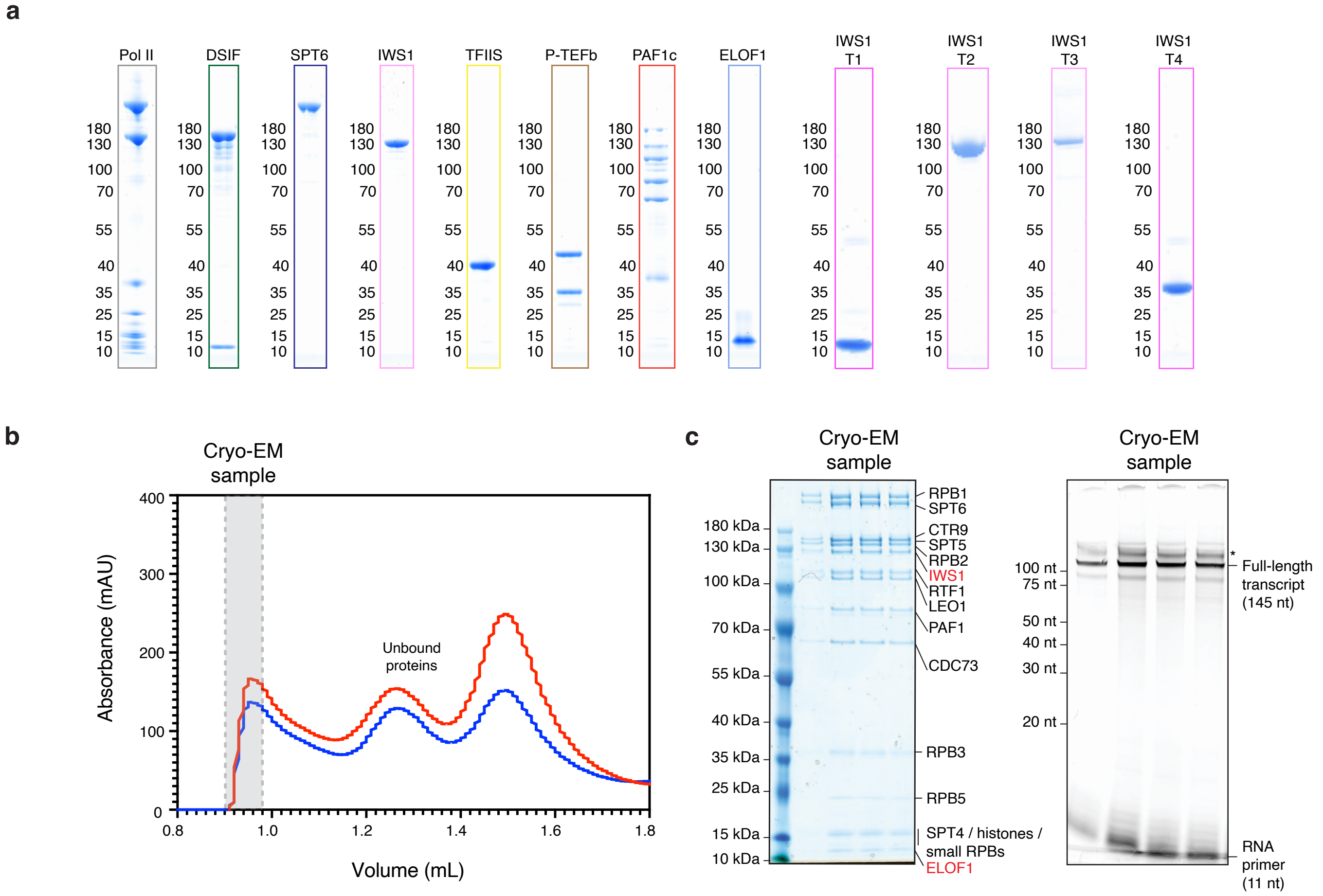

Supplementary Fig. 3. Protein purity and complex assembly.

a) SDS-PAGE analysis of purified proteins. Molecular weights are indicated.

b) Chromatogram of Superose 6 Increase (3.2/300) purification of the complex used for cryo-EM. Frozen fractions are indicated in gray. Red and blue traces indicate absorbance at 260 and 280 milli-absorbance units, respectively.

c) Left: SDS-PAGE of fractions used for cryo-EM. Protein components of the complex are marked. Right: 12% TBE-urea PAGE of fractions used for cryo-EM. Full-length transcript and RNA primer are marked. Asterisk indicates non-denatured nucleic acid hybrid.

Supplementary Fig. 4

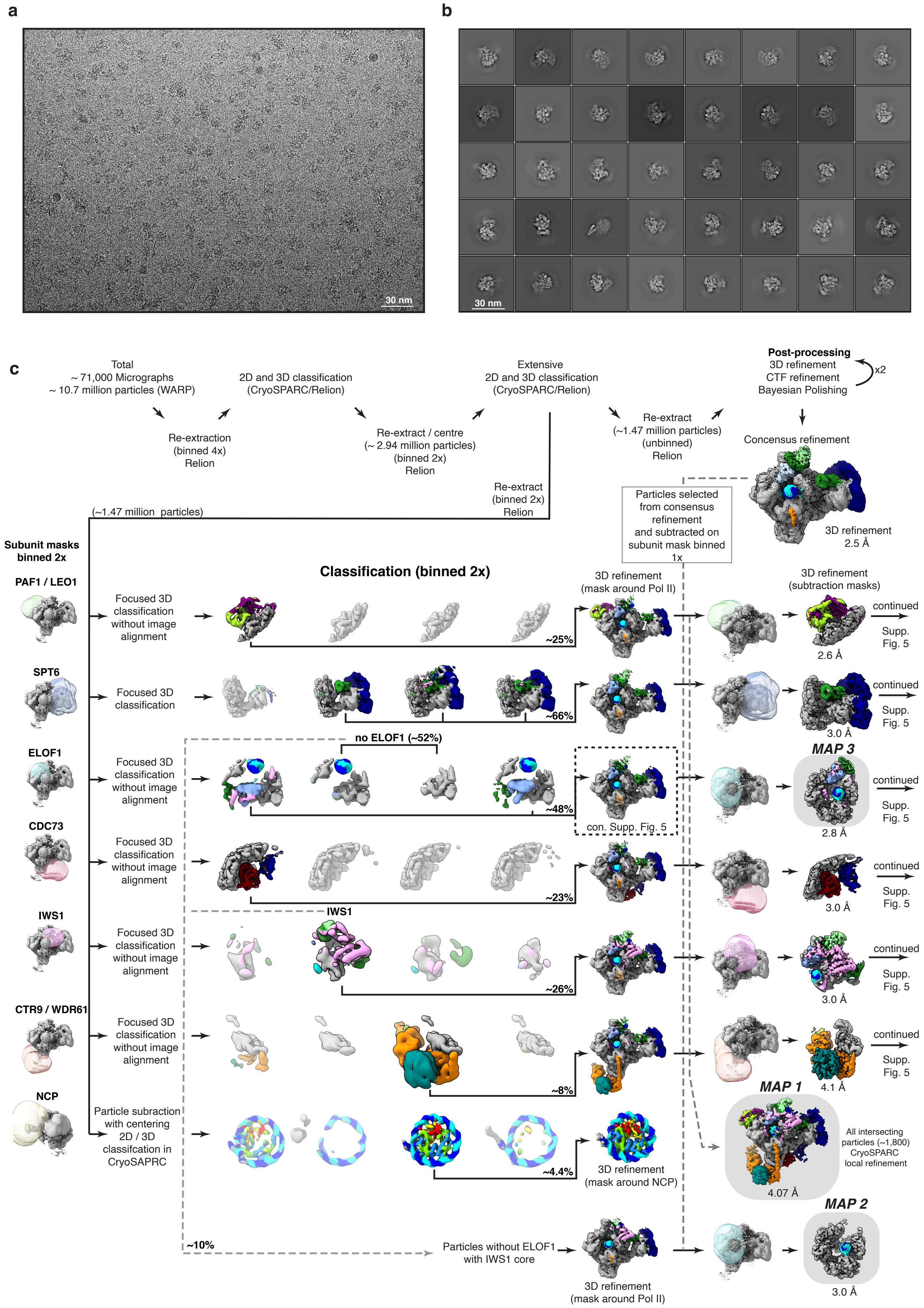

Supplementary Fig. 4

Supplementary Fig. 4. Data acquisition and initial processing of cryo-EM data of the activated elongation complex with IWS1 and ELOF1.

a) Representative micrograph of data collection with scale bar.
b) Representative 2D classes of activated elongation complex with IWS1 and ELOF1 obtained during classification.
c) Processing and classification strategy employed to initially classify occupancy of elongation factors associated with the activated elongation complex with IWS1 and ELOF1. Major steps are described within the figure. Cryo-EM maps used for model building and analysis are highlighted in gray.

Supplementary Fig. 5

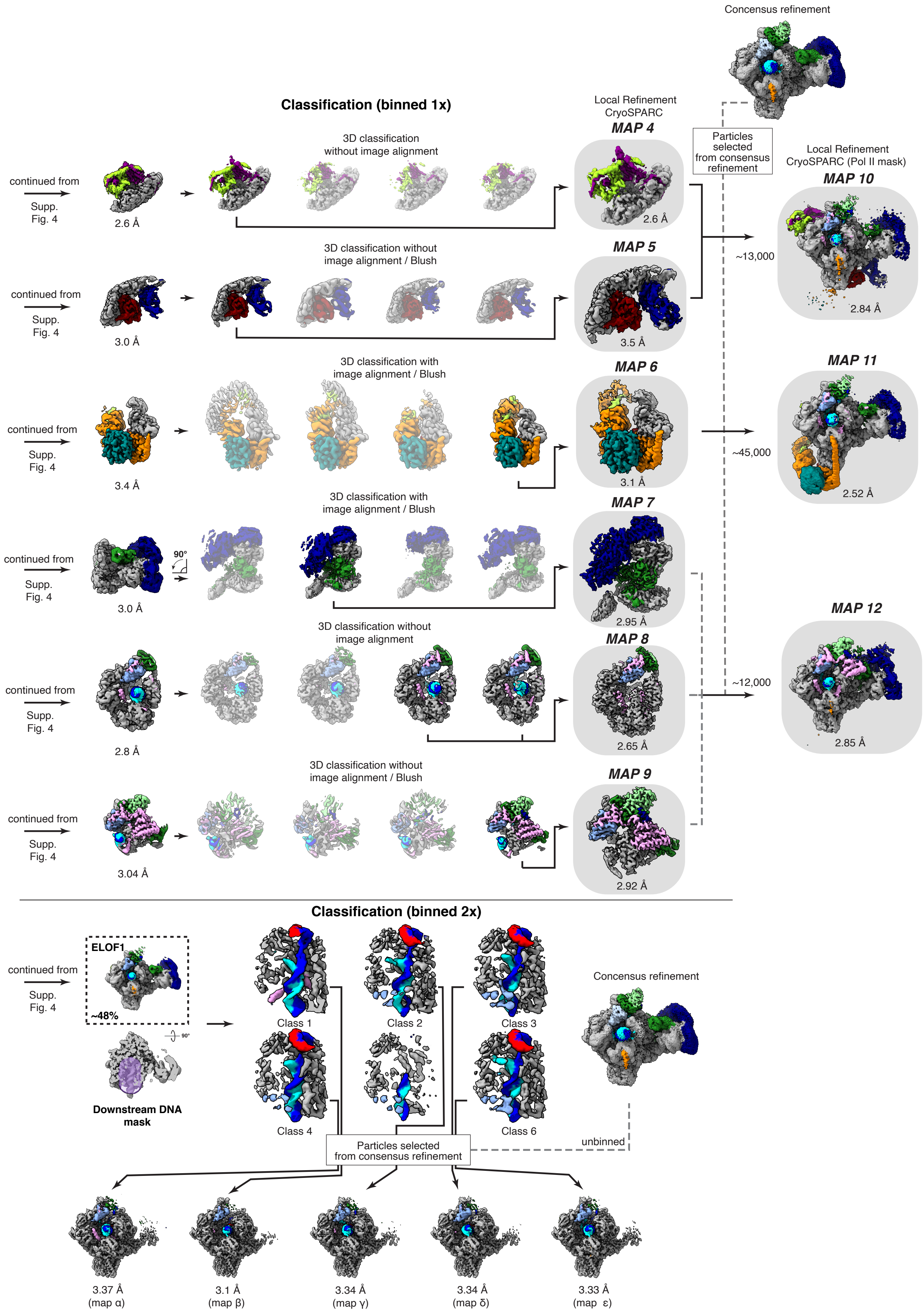

Supplementary Fig. 5

Supplementary Fig. 5. Final processing of cryo-EM data of the activated elongation complex with IWS1 and ELOF1.

Final processing and classification strategy employed to obtain the best resolution reconstructions of components of the activated elongation complex with IWS1 and ELOF1. Major steps are described within the figure. Cryo-EM maps used for model building are highlighted in gray.

Supplementary Fig. 6

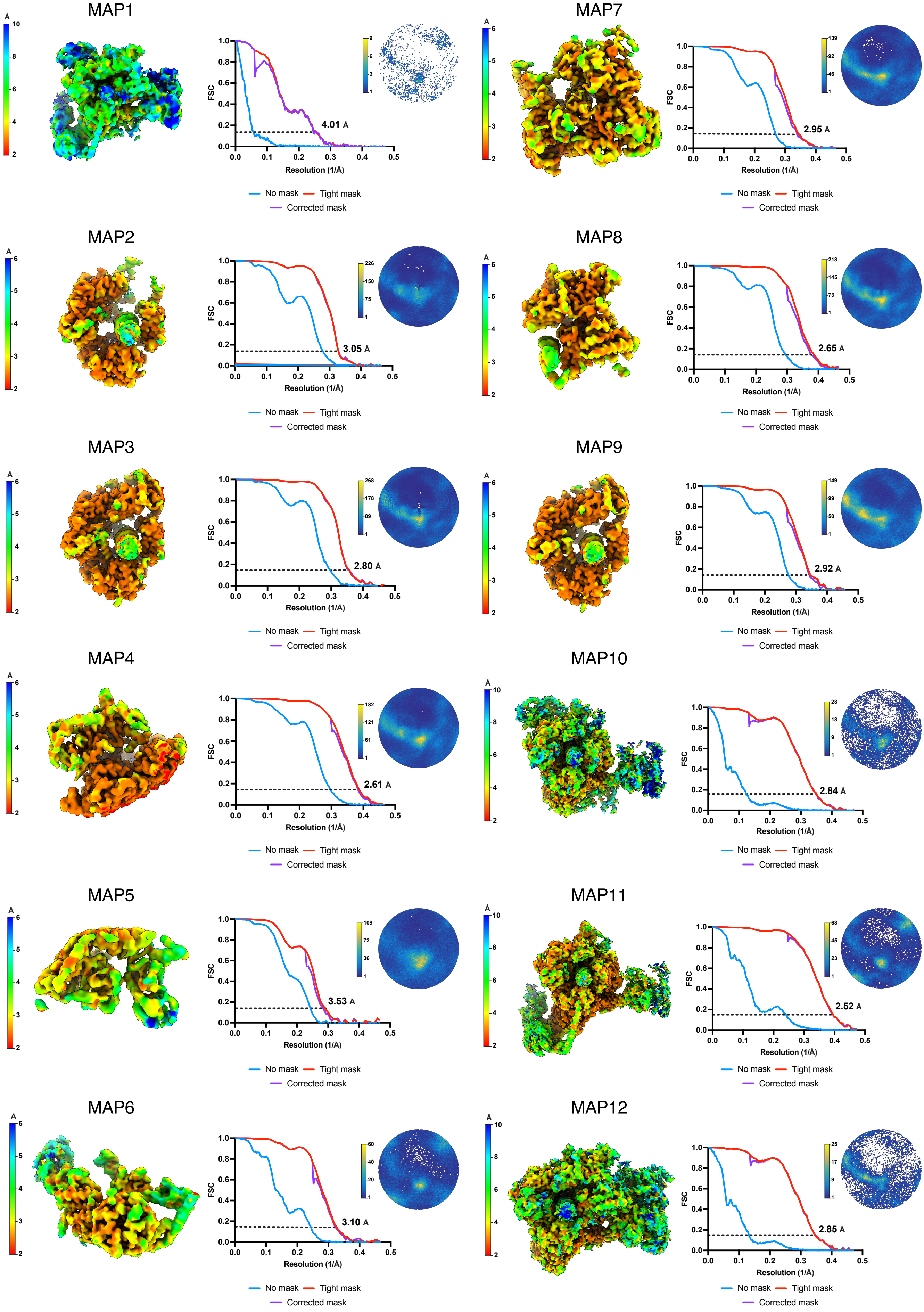

Supplementary Fig. 6

Supplementary Fig. 6. Local resolution estimation.

Final reconstructions obtained from classification of the cryo-EM data of the activated elongation complex with IWS1 and ELOF1, colored by local resolution as estimated using CryoSPARC (74). The global resolution was determined using the Fourier shell correlation (FSC) criterion at 0.143 with corresponding plots shown. The angular distribution for particles in the final reconstructions are shown. Shading from blue to yellow correlates with increasing number of particles at a given orientation.

Supplementary Fig. 7

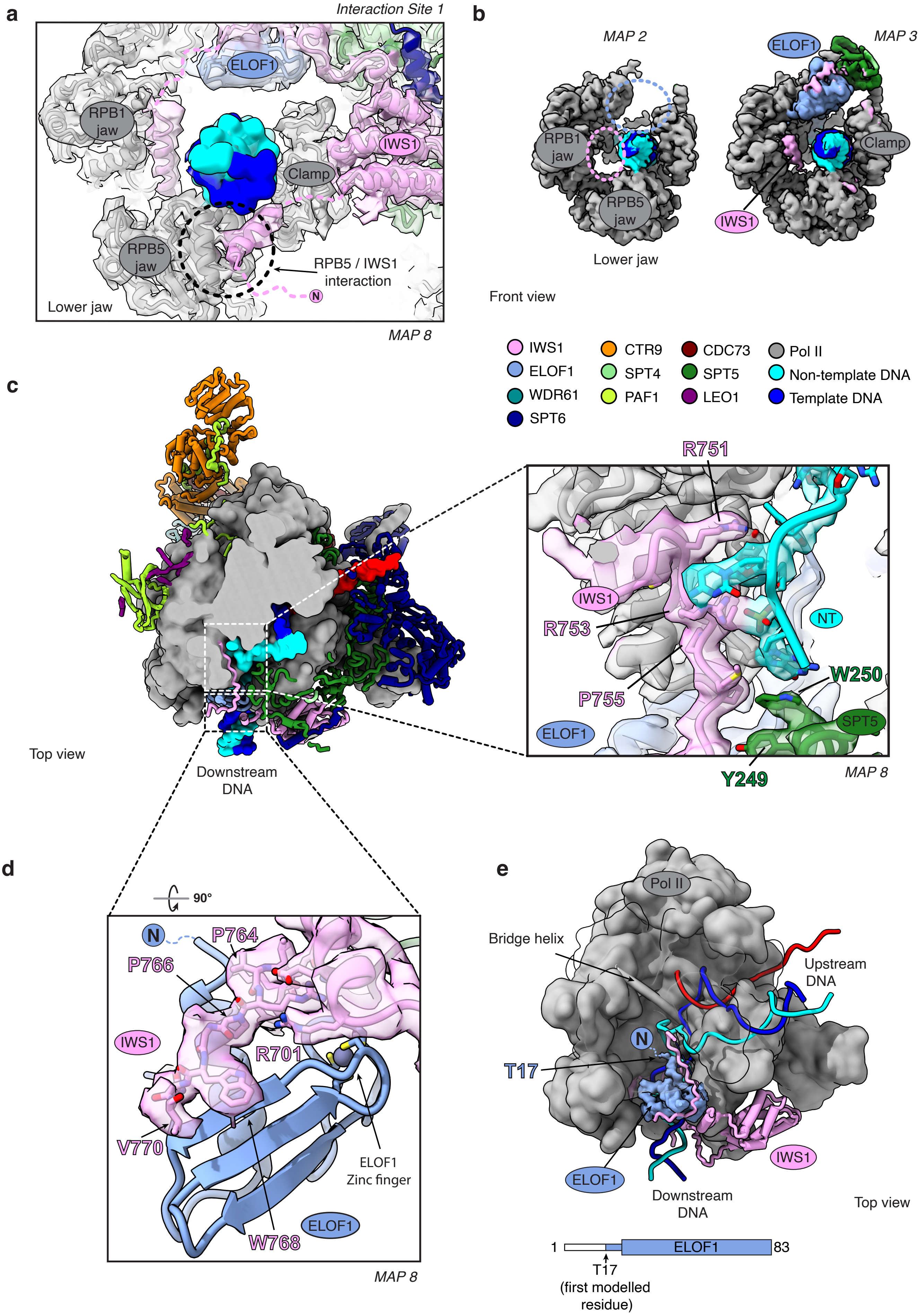

Supplementary Fig. 7. ELOF1 cooperates with the ECS domain of IWS1.

a) Interaction Site 1: Residues 528-539 of the ECS domain of IWS1 contacts the lower jaw of RPB5. Unmodelled N-terminal of IWS1 represented as dashes. Cryo-EM MAP8 shown transparent and colored as per figure legend.
b) Front view of cryo-EM reconstructions corresponding to MAP2 and MAP3 colored as in a). Absence of cryo-EM density representing ELOF1 and the C-terminal of IWS1 in MAP2 indicated as dashed circles.
c) Sliced top view of the activated elongation complex with IWS1 and ELOF1. Zoomed-in panel showing alternative view of Interaction Site 3 with non-template DNA strand modeled. Cryo-EM MAP8 shown transparent and colored as per figure legend.
d) Zoomed-in panel showing alternative view of Interaction Site 3. ELOF1 zinc atom is shown as gray sphere and with cysteine residues shown in stick representation. Cryo-EM MAP8 shown transparent and colored as per figure legend.
e) Top view of activated elongation complex with IWS1 and ELOF1. N-terminal of ELOF1 points towards the bridge helix of Pol II. Elongation factors, with the exception of ELOF1 and IWS1, are hidden for clarity. 2D schematic of modelled ELOF1 residues are shown in pale blue.

Supplementary Fig. 8

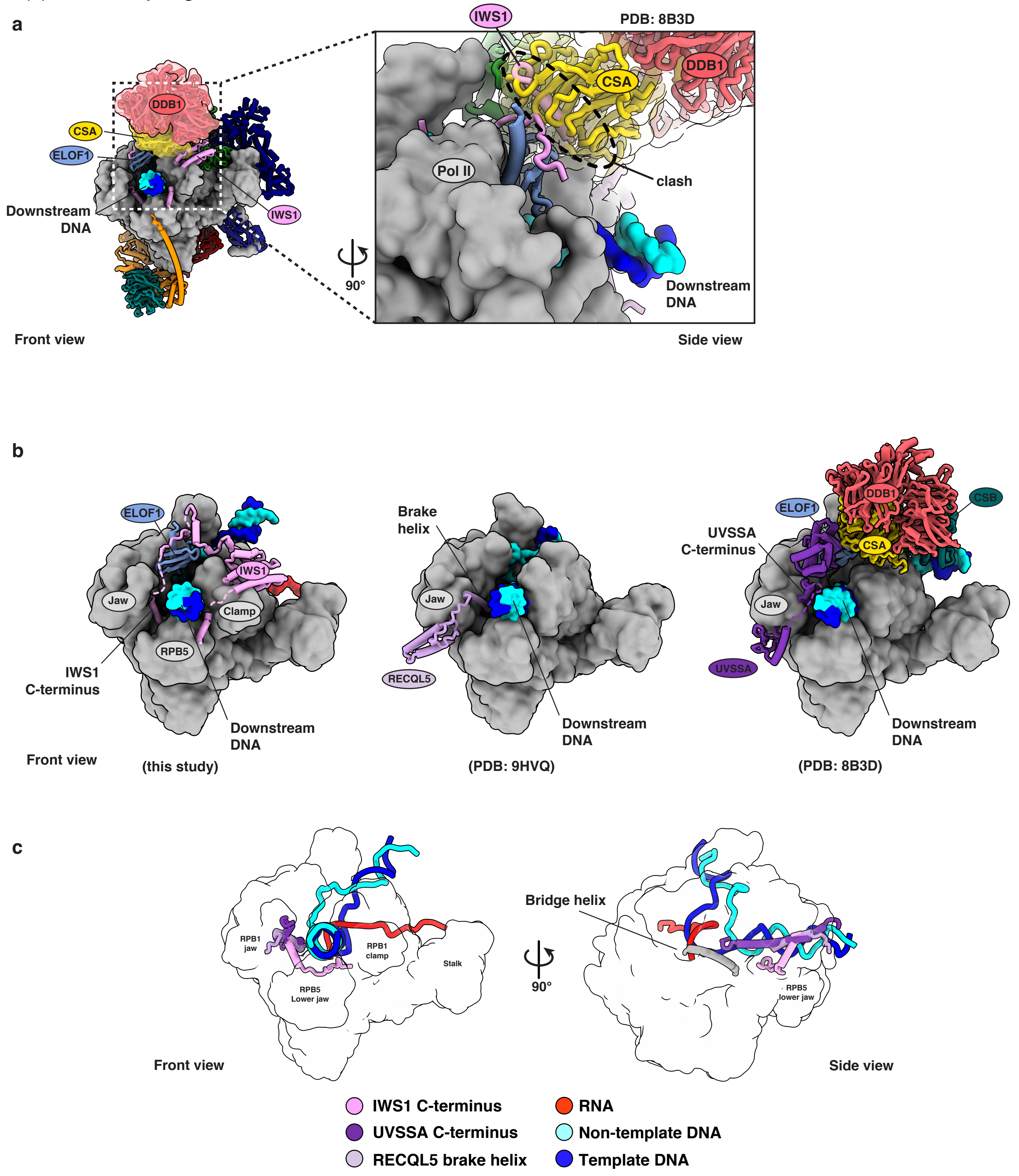

Supplementary Fig. 8. IWS1 comparison RECQL5, UVSSA and CSA.

a) Structural overlay of the activated elongation complex with IWS1 and ELOF1 and the Pol II–TCR–ELOF1 complex (PDB 8B3D). Cockayne syndrome A (CSA) ubiquitin ligase and IWS1 bind the same interface of ELOF1 (dashed circle).

b) Structural comparison of the activated elongation complex with IWS1 and ELOF1 (only Pol II subunits, ELOF1 and IWS1 visualized), the Pol II-RECQL5 complex (PDB: 9HVQ) and the Pol II–TCR–ELOF1 complex (PDB 8B3D). Components of each complex access the Pol II cleft via the jaw of RPB1.

c) Front and side view of overlayed complexes shown in b) showing the regions that interact via the Pol II jaw.

Supplementary Fig. 9

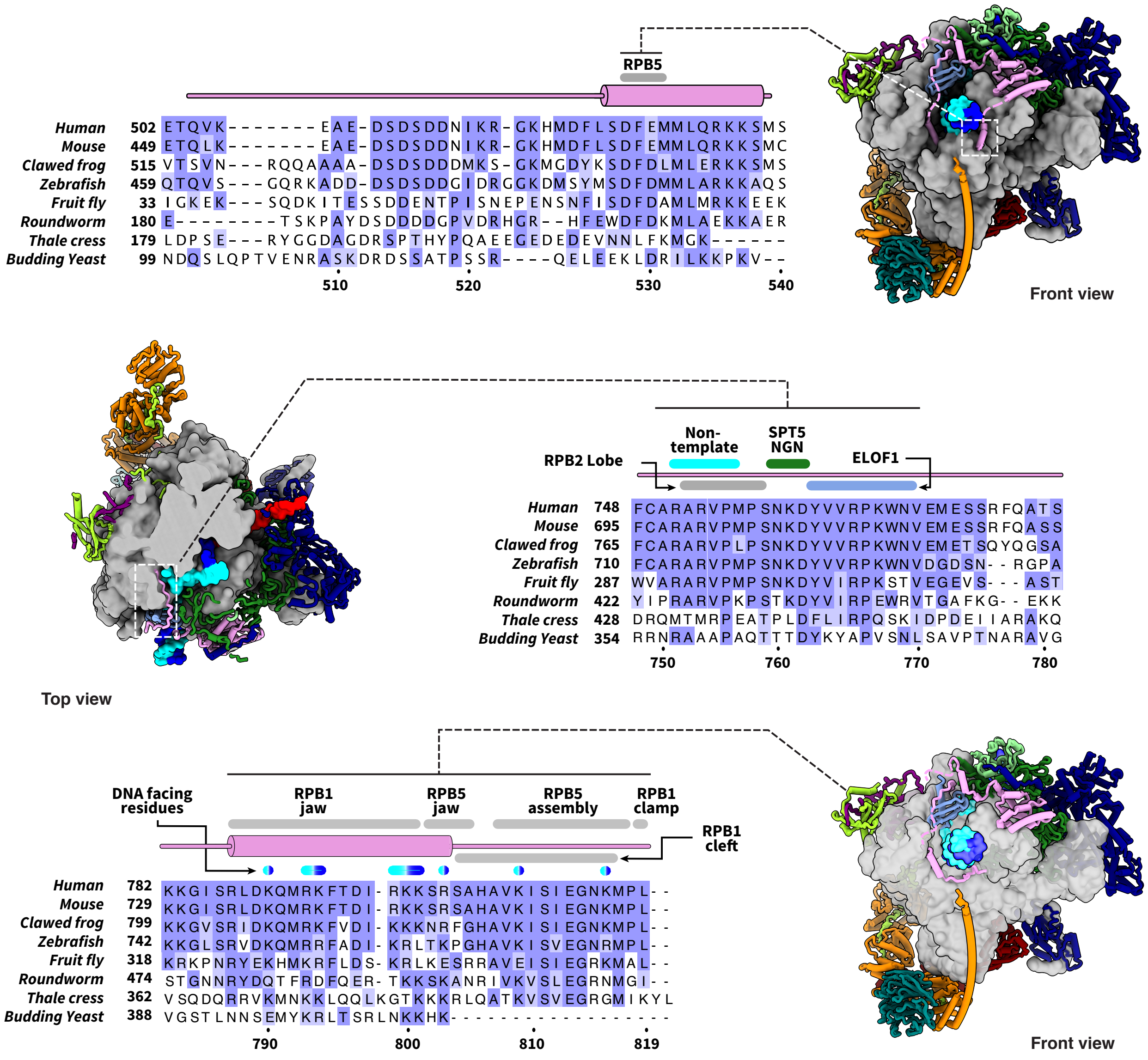

Supplementary Fig. 9. ECS domain of IWS1 sequence alignment.

Sequence alignment of human IWS1 and homologues from Mouse, Clawed frog, Zebrafish, Fruitfly, Roundworm, Thale cress, and budding yeast corresponding to Uniport IDs: Q96ST2, Q8C1D8, Q6DE96, A0A8M1RRH3, B4F5R2, Q19375, F4ICK8, Q06505, respectively. Sequence alignment generated using ClustalOmega (78) and visualised using Jalview (79). Predicted secondary structure is shown above the sequences and interaction regions with Pol II, DNA and elongation factors are indicated.

Supplementary Fig. 10

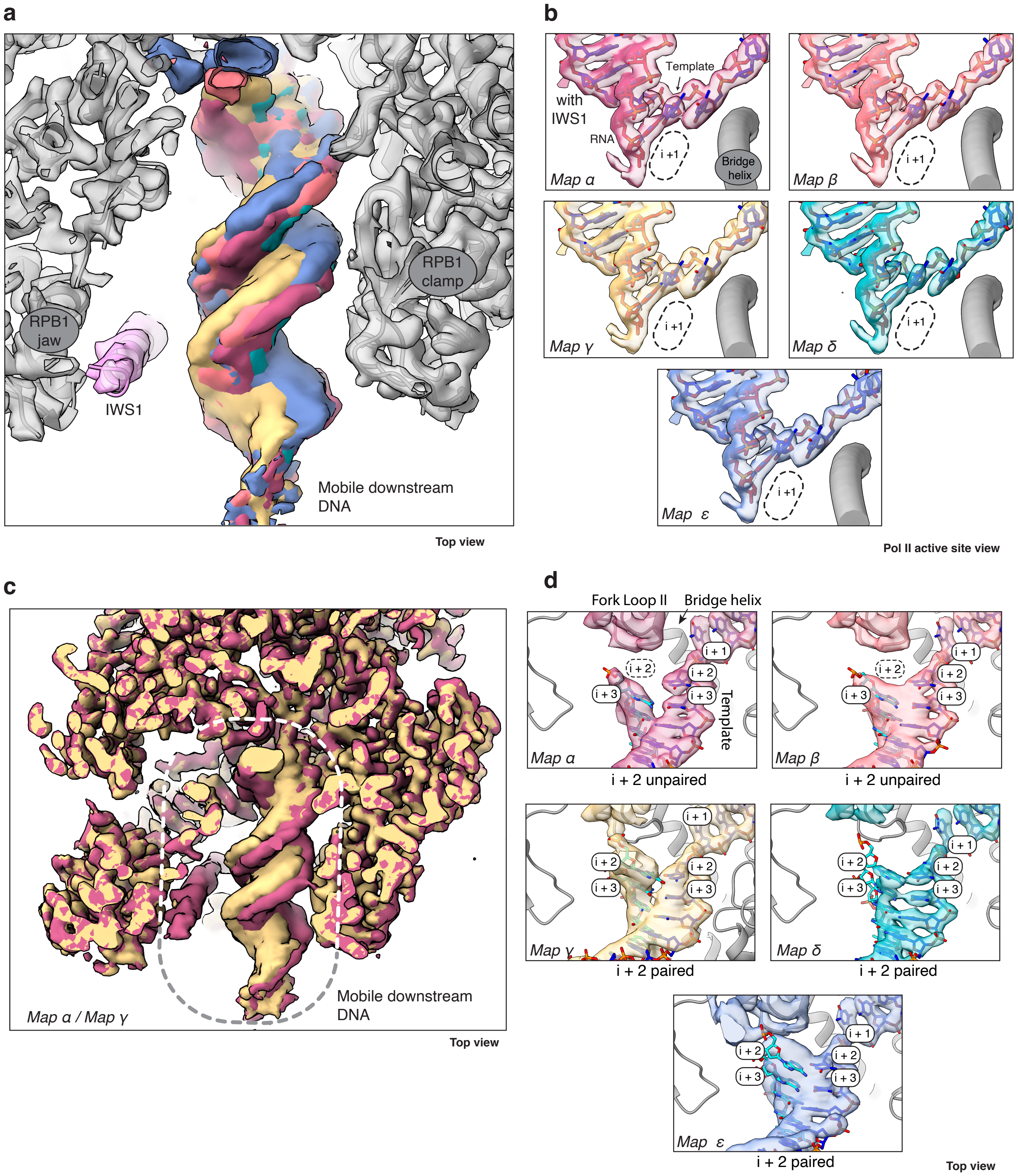

Supplementary Fig. 10. IWS1 limits DNA mobility in the Pol II cleft.

a) Comparison of the cryo-EM densities corresponding to the downstream DNA within the Pol II cleft of MAP α, MAP β, MAP γ, MAP δ, MAP ε (Supplementary Fig. 5). Only MAP α contains density representing the C-terminal of the ECS domain. Density for RPB1 and IWS1 from MAP α coloured in gray and pink respectively.

b) Pol II active site view of MAP α, MAP β, MAP γ, MAP δ, MAP ε showing the cryo-EM density corresponding to the DNA / RNA hybrid. In all maps Pol II adopts a post-translocated state.

c) Comparison of the cryo-EM densities of MAP α and MAP γ.

d) Pol II Top view of MAP α, MAP β, MAP γ, MAP δ, MAP ε showing the cryo-EM density representing the downstream DNA. Unpairing of the downstream DNA observed at the i + 2 position in MAP α (with IWS1) and MAP β.

Supplementary Fig. 11

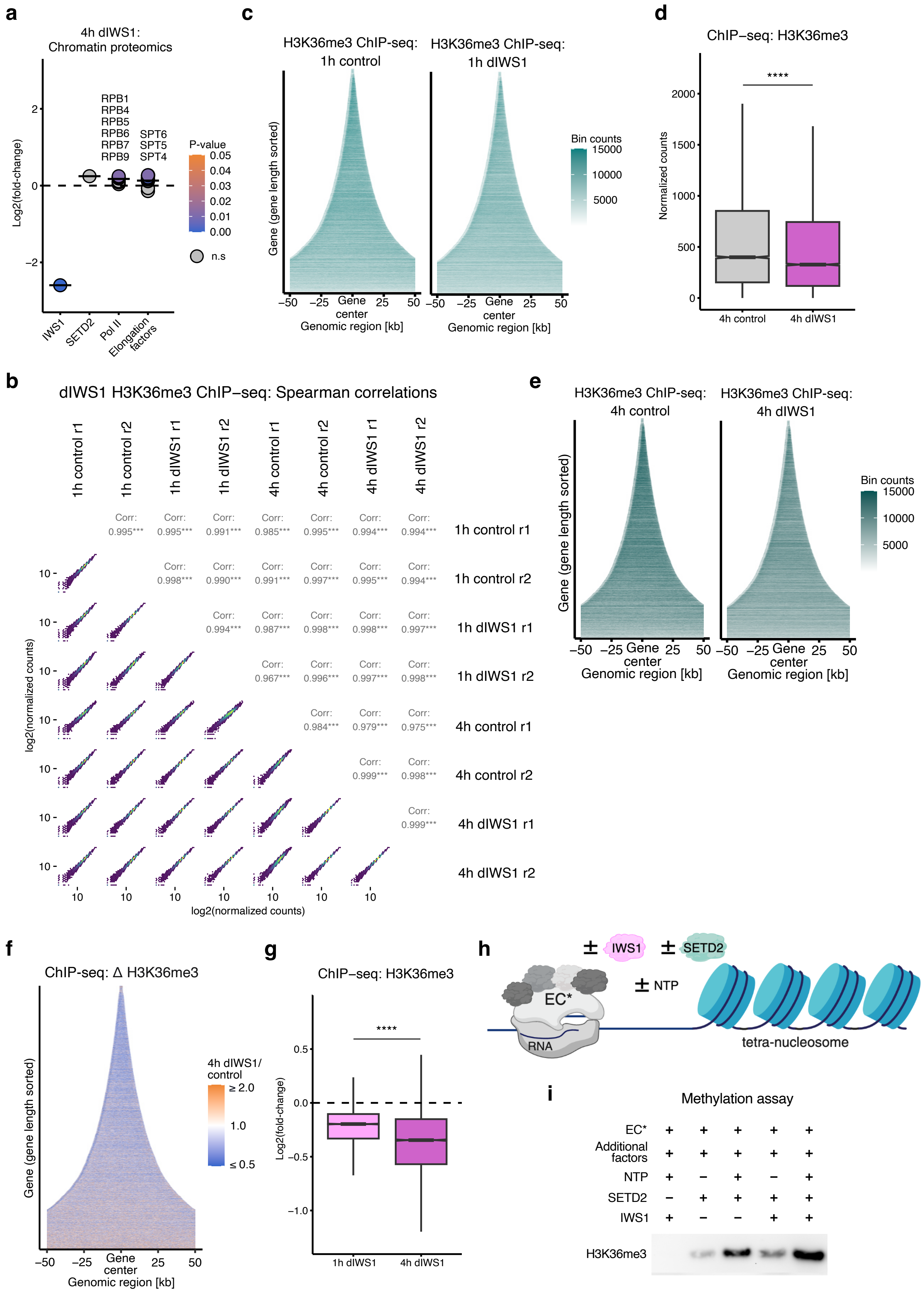

Supplementary Fig. 11

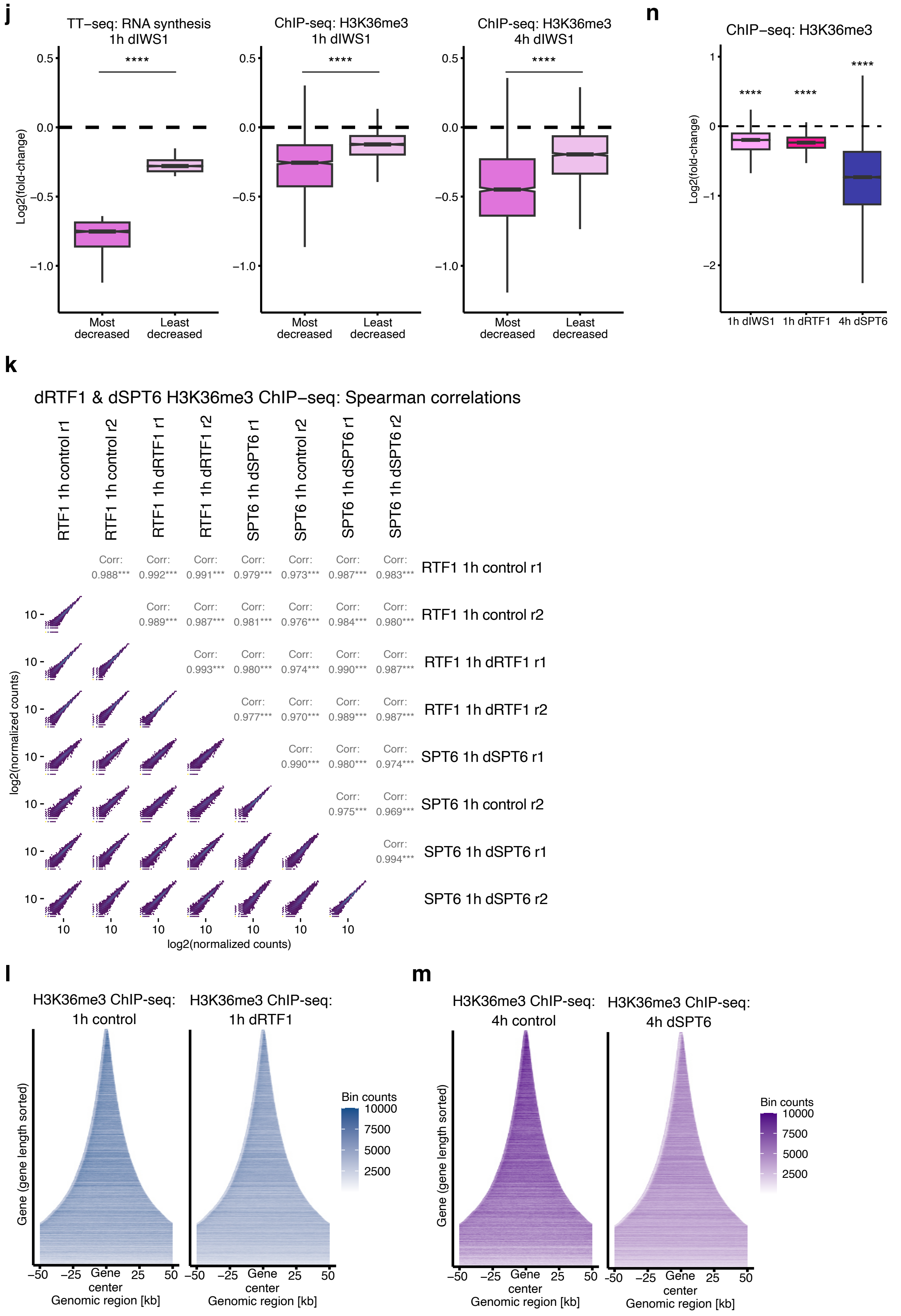

Supplementary Fig. 11

Supplementary Fig. 11. IWS1 stimulates Pol II elongation to support H3K36me3.

a) As in Fig. 5d, but after 4 h depletion of IWS1.
b) Scatter plots of normalized H3K36me3 ChIP-seq gene counts and Spearman correlation coefficients (rho) of all sample pairs (n=10147).
c) Heatmap representation of H3K36me3 ChIP-seq signal of expressed genes (n=10147) aligned at the gene centers in control- or dTAG7-treated (dIWS1) cells. Representation as in Fig. 2b.
d) As in Fig. 5e, but after 4 h depletion of IWS1. p-value $< 2.2*10^{-16}$.
e) As in c), but after 4 h depletion of IWS1.
f) As in Fig. 5f, but after 4 h depletion of IWS1.
g) Boxplots show fold-changes in H3K36me3 ChIP-seq counts after 1 h and 4 h depletions of IWS1 in expressed genes (n=10147). Representations as in Fig. 2e. p-value $< 2.2*10^{-16}$.
h) Schematic representation of the in vitro methylation assay with the complete activated elongation complex (Pol II, DSIF, PAF1 complex, RTF1, SPT6) and additional factors (see Methods). The assay was performed on a tetra-nucleosome array. Created in BioRender. Zheenbekova, A. (2025) https:// BioRender.com/0lp88l0
i) Western blot analysis of H3K36me3 methylation assay depicted in h).
j) Boxplots show fold-change in RNA synthesis (TT-seq) after 1 h depletion of IWS1 and H3K36me3 ChIP-seq after 1h and 4 h depletions of IWS1 for gene subgroups, where the "Most decreased" group represents the top 25% of significantly down-regulated genes (n=1876), while the "Least decreased" group represents the bottom 25% of significantly down-regulated genes (n=1876) based on TT-seq data. Representation as in Fig. 2e. TT-seq: p-value $< 2.2*10^{-16}$, H3K36me3 ChIP-seq 1h: p-value $< 2.2*10^{-16}$, H3K36me3 ChIP-seq 4h: p-value $< 2.2*10^{-16}$.
k) Scatter plots of normalized H3K36me3 ChIP-seq gene counts after 1 h RTF1 and 4 h SPT6 depletions and Spearman correlation coefficients (rho) of all sample pairs (n=23036).
l) As in c), but after 1 h depletion of RTF1 (n=12976).
m) As in c), but after 4 h depletion of SPT6 (n=14002).
n) As in g), but for 1 h IWS1 (n=10147), 1 h RTF1 (n=12976) and 4 h SPT6 (n=14002) depletions. IWS1: p-value $< 2.2*10^{-16}$, RTF1: p-value $< 2.2*10^{-16}$, SPT6: p-value $< 2.2*10^{-16}$.

Supplementary Table 1. Oligonucleotide sequences

Name	Sequence	Note
**sgIWS1_N1**	gagtccgcctgccatggact	sgRNA
**mHD5'_IWS1_N1_F**	gcgttacatagcatcgtacgcgtacgtgtttggtgagagtccgc<br>ctgccatggtgaccgagtacaagcccacg	HR template cloning primer; underlined = microhomology + frame correction
**mHD3'_IWS1_N1_R**	agcattctagagcatcgtacgcgtacgtgtttgggccgctgtaat<br>attccgagtcagatccgccgccacc	HR template cloning primer; underlined = microhomology + frame correction
**IWS1_N_F**	gggtcattagttcatagccc	Genotyping primer
**IWS1_N_R**	tattaggaaaggacagtggg	Genotyping primer
**RNA primer for transcription assay**	/5Cy5/UUAUCACUGUC	5' Cy5 labelled
**Template strand *in vitro* transcription assay**	ATCAGAATCCCGGTGCCGAGGCCGCTCAA<br>TTGGTCGTAGACAGCTCTAGCACCGCTTAA<br>ACGCACGTACGCGCTGTCCTTTTCGAAAAA<br>ACCGCCAAGGGGAAAACACCCAAGACACC<br>AGGCACGAGACAGAAAAAAACAACGAAAA<br>CGGCCACCACCCAAGCAGCGCAGGACGA<br>CGAGCGGACCCACCACCAACAACCAAAAC<br>ACACCAAACACAAGACAGTGAT	*In vitro* transcription DNA template; underlined = primer binding site
**Template_amplification_R *in vitro* transcription**	GCAGTCCAGTTACGCTGGAGTC	*In vitro* transcription template
**Template_amplification_F *in vitro* transcription**	ATCAGAATCCCGGTGCCG	*In vitro* transcription template amplification primer
**Template strand *in vitro* methylation**	ATCGATCCCCTGGAGAATCCCGGTGCCGA<br>GGCCGCTCAATTGGTCGTAGACAGCTCTA<br>GCACCGCTTAAACGCACGTACGCGCTGTC<br>CCCCGCGTTTTAACCGCCAAGGGGATTAC<br>TCCCTAGTCTCCAGGCACGTGTCACATATA<br>TACATCCTGTCTTGAAAGCGCATTTAAATC<br>ATACAACTGCCTGGAGAATCCCGGTGCCG<br>AGGCCGCTCAATTGGTCGTAGACAGCTCT<br>AGCACCGCTTAAACGCACGTACGCGCTGT<br>CCCCCGCGTTTTAACCGCCAAGGGGATTA<br>CTCCCTAGTCTCCAGGCACGTGTCACATAT<br>ATACATCCTGTCATGATGCGTACAGATCTT<br>CCAGTACACCGCTGGAGAATCCCGGTGCC<br>GAGGCCGCTCAATTGGTCGTAGACAGCTC<br>TAGCACCGCTTAAACGCACGTACGCGCTG<br>TCCCCCGCGTTTTAACCGCCAAGGGGATT<br>ACTCCCTAGTCTCCAGGCACGTGTCACATA<br>TATACATCCTGTTCTCTGTAGGCCGGATCC<br>AAGAGCTGGAAGCTGGAGAATCCCGGTGC<br>CGAGGCCGCTCAATTGGTCGTAGACAGCT<br>CTAGCACCGCTTAAACGCACGTACGCGCT<br>GTCCCCCGCGTTTTAACCGCCAAGGGGAT<br>TACTCCCTAGTCTCCAGGCACGTGTCACAT<br>ATATACATCCTGTTTCTGCCGTTGTGGCGT<br>TGTTCTCTTCCTCGCCCAGACAGTGATGCT<br>GCGAT	*In vitro* methylation template; underlined = primer binding site
**Template strand cryoEM complex**	ATCAGAATCCCGGTGCCGAGGCCGCTCAA<br>TTGGTCGTAGACAGCTCTAGCACCGCTTAA<br>ACGCACGTACGCGCTGTCCCCCGCGTTTT<br>AACCGCCAAGGGGATTACACCTTTTACACC<br>AGGCACGAGACAGAAAAAAACAACGAAAA	DNA template used for cryoEM complex assembly; underlined = primer binding site

	CGGCCACCACCCAAGCAGCGCAGGACGA<br>CGAGCGGAACCCAACCACCACCACAAACC<br>AACCACACACCCACCACCAACAACCAAAAC<br>ACACCAAACACAAGACAGTGATG	
**Template_amplification_F cryoEM complex**	ATCAGAATCCCGGTGCCG	Cryo-EM complex transcription template amplification primer
**Template_amplification_R cryoEM complex**	ACGAAGCGTAGCATCACTGTCTTG	Cryo-EM complex transcription template amplification primer

Supplementary Table 2. Cryo-EM data collection, refinement and validation statistics

Activated elongation complex with ELOF and IWS1
EMDB-54251 – **MAP1**
(PDB 9RTT)

**Data collection and processing**	
Magnification	81 000x
Voltage (kV)	300
Electron exposure (e–/Å^2)	40.03
Defocus range (μm)	0.3-1.8
Pixel size (Å)	1.05
Initial particle images (no.)	1,478,120
Final particle images (no.)	1,837
Map resolution (Å)	4.07
FSC threshold	0.143

	**MAP2**	**MAP3**	**MAP4**	**MAP5**	**MAP6**	**MAP7**
EMDB	54196	54197	54201	54202	54203	54205
Final particle images (no.)	154171	213724	175394	103931	45083	118496
Map resolution (Å)	3.05	2.80	2.61	3.53	3.1	2.95
FSC threshold	0.143	0.143	0.143	0.143	0.143	0.143
Map sharpening *B* factor (Å^2)	-94.9	-96.5	-79.6	-138.8	-61.5	-75.7

	**MAP8**	**MAP9**	**MAP10**	**MAP11**	**MAP12**
EMDB	54206	54207	54208	54209	54210
Final particle images (no.)	213724	176056	13111	45083	12626
Map resolution (Å)	2.65	2.92	2.84	2.52	2.85
FSC threshold	0.143	0.143	0.143	0.143	0.143
Map sharpening *B* factor (Å^2)	-86.5	-100.2	-25.2	-37.4	-29.5

Refinement and validation

Initial model used (PDB code)	6TED		
Map sharpening *B* factor (Å^2)	-197.5		
Model composition		Validation	
Non-hydrogen atoms	64,867	MolProbity score	1.66
Protein residues	7,816	Clashscore	3.51
Nucleotide residues	95	Poor rotamers (%)	1.97
Ligands	10		
B factors (Å^2)		Ramachandran plot	
Protein	121.99	Favored (%)	95.80
Nucleotide	190.21	Allowed (%)	4.11
Ligand	134.01	Disallowed (%)	0.09
R.m.s. deviations			
Bond lengths (Å)	0.007		
Bond angles (°)	1.457		
